# NTFP harvesters as citizen scientists: Validating traditional and crowdsourced knowledge on seed production of Brazil nut trees in the Peruvian Amazon

**DOI:** 10.1371/journal.pone.0183743

**Published:** 2017-08-24

**Authors:** Evert Thomas, Jheyson Valdivia, Carolina Alcázar Caicedo, Julia Quaedvlieg, Lucia Helena O. Wadt, Ronald Corvera

**Affiliations:** 1 Bioversity International, Lima, Peru; 2 Universidad Nacional Amazonica de Madre de Dios, Puerto Maldonado, Peru; 3 Bioversity International, Cali, Colombia; 4 Independent consultant, Puerto Maldonado, Peru; 5 Embrapa Rondônia, Brazil; 6 Instituto de Investigación para la Amazonıa Peruana, Puerto Maldonado, Peru; New York State Museum, UNITED STATES

## Abstract

Understanding the factors that underlie the production of non-timber forest products (NTFPs), as well as regularly monitoring production levels, are key to allow sustainability assessments of NTFP extractive economies. Brazil nut (*Bertholletia excelsa*, Lecythidaceae) seed harvesting from natural forests is one of the cornerstone NTFP economies in Amazonia. In the Peruvian Amazon it is organized in a concession system. Drawing on seed production estimates of >135,000 individual Brazil nut trees from >400 concessions and ethno-ecological interviews with >80 concession holders, here we aimed to (i) assess the accuracy of seed production estimates by Brazil nut seed harvesters, and (ii) validate their traditional ecological knowledge (TEK) about the variables that influence Brazil nut production. We compared productivity estimates with actual field measurements carried out in the study area and found a positive correlation between them. Furthermore, we compared the relationships between seed production and a number of phenotypic, phytosanitary and environmental variables described in literature with those obtained for the seed production estimates and found high consistency between them, justifying the use of the dataset for validating TEK and innovative hypothesis testing. As expected, nearly all TEK on Brazil nut productivity was corroborated by our data. This is reassuring as Brazil nut concession holders, and NTFP harvesters at large, rely on their knowledge to guide the management of the trees upon which their extractive economies are based. Our findings suggest that productivity estimates of Brazil nut trees and possibly other NTFP-producing species could replace or complement actual measurements, which are very expensive and labour intensive, at least in areas where harvesters have a tradition of collecting NTFPs from the same trees over multiple years or decades. Productivity estimates might even be sourced from harvesters through registers on an annual basis, thus allowing a more cost-efficient and robust monitoring of productivity levels.

## Introduction

The integration of crowdsourcing approaches in citizen science has recently become a mayor tool to collect and analyse large quantities of data efficiently and often cheaply, not only in the traditional areas of conservation biology [1], but also increasingly in scientific domains related to plant production and improvement [2]. While the possible applications of crowdsourcing are vast, a valid concern relates to the trade-off between involving increasing numbers of people and ensuring data quality, particularly so when consulting laypersons on specialized knowledge domains. Assessments of data quality from citizen science are on the rise. Most studies have focused on species observations within the ambit of conservation biology, where the ability of laypeople to recognize and discriminate between species sets the boundaries to the level of detail at which data can be sourced with an acceptable degree of accuracy and reliability [3,4]. Assessments of the quality of crowdsourced productivity data at the intraspecific level have received far less attention until present [5].

Here we assess the accuracy of seed production estimates of the Brazil nut (*Bertholletia excelsa*, Lecythidaceae), one of the most important non-timber forest products in Amazonia [6], by people engaging in annual seed harvesting in the Peruvian Amazon. Experimental appraisals of the average seed production of individual Brazil nut trees in natural forests can be very complex and costly. Measurements necessarily have to run over several years to account for the high interannual variability in fruit production [7]. Due to these limitations, studies that investigated productivity of Brazil nut have considered a limited number of trees (<500) [8–12]. An alternative approach is to source productivity data estimates from Brazil nut harvesters [10]. Forest-dependent people often have an intimate knowledge of the natural resources they harvest and use, suggesting that they can be reliable sources of good quality citizen science data. The *savoir faire* of forest dwellers is generally referred to as traditional ecological knowledge (TEK) which can be defined as “a cumulative body of knowledge, know-how, practices and representations maintained and developed by peoples with extended histories of interaction with the natural environment” [13].

The Peruvian Brazil nut sector is organized in a family-managed 40-year concession system, which was established in response to the Peruvian Forestry Law N°27308 (16/7/2000) as a way of formalizing traditional usufruct rights [9]. Concessionaires in the Peruvian Amazon department of Madre de Dios either collect Brazil nut seeds themselves, or they hire people for this purpose. In what follows we will refer to the people directly involved in seed collection as Brazil nut harvesters. Most Brazil nut harvesters in Madre de Dios have been collecting seeds for decades [14] and can be expected to hold a realistic notion of the average productivity of individual trees in their concessions. For example, Wadt et al [10] found that in the nearby Brazilian state of Acre, Brazil nut harvesters’ average annual nut production estimates of individual trees based on their recollection from the previous 5-year collection period were closely aligned with measured production levels. Furthermore, harvesters hold a vast body of TEK on the biological and anthropogenic factors that may influence seed production of Brazil nut. Drawing on harvesters’ estimates of the average seed production of >135,000 individual Brazil nut trees, our purpose here is dual. First, we aim to validate the usefulness of local harvesters’ estimates of average seed production of individual trees as proxies for actual measurements. We do this by comparing (i) production estimates with field measurements carried out in the study area by Rockwell et al. [9] for a subset of the trees with seed production estimates and (ii) the relationships between seed production and a number of phenotypic, phytosanitary and environmental variables described in literature with those found for our own data. Second, where possible, we used the seed productivity estimates to test the validity of TEK on variables that influence productivity, based on interviews with a selected set of Brazil nut concessionaires. Furthermore, we discuss the relationships observed between a number of additional spatial habitat variables and Brazil nut seed production estimates to identify future avenues for research.

## Materials and methods

### Data collection

**Fig 1:**
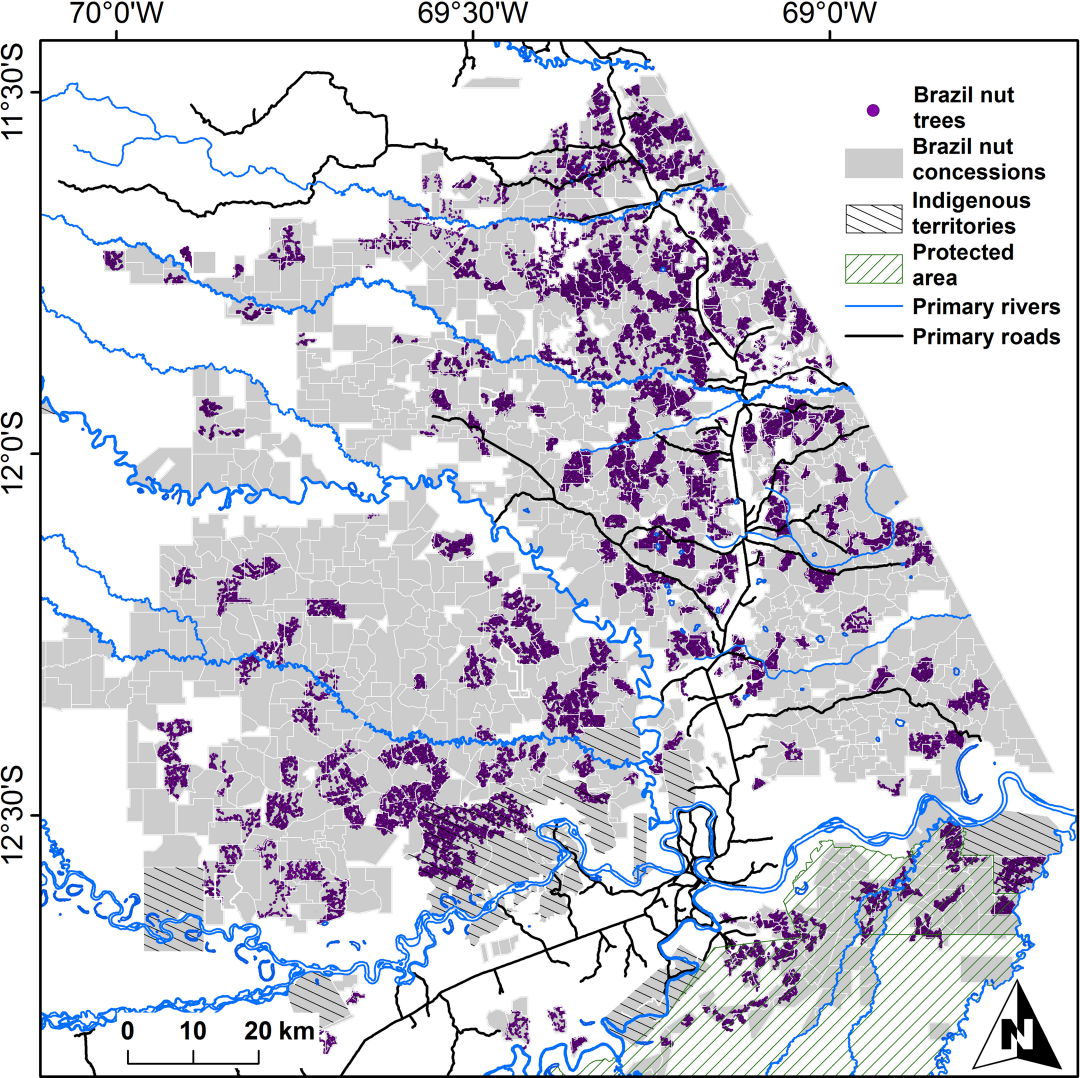
Distribution of Brazil nut concessions in Madre de Dios, Peru, with approximate locations of the 135,528 individual Brazil nut trees (DBH≥10 cm) from 418 concessions considered in this paper.

The Peruvian Forestry Law N°27308 obliged concession holders for the first time to present detailed inventories of the Brazil nut trees under their custody. Most inventories were carried out under the auspices of a number of institutions active in the region, most notably ACCA (Asociación para la Conservación de la Cuenca Amazónica), CAMDE (Conservación Ambiental y Desarrollo en el Perú), FONDEBOSQUE (Fondo de Promoción del Desarrollo Forestal), AIDER (Asociación para la Investigación y Desarrollo Integral), RNTAMB PRMRFFS (Programa Regional de Manejo de Recursos Forestales y Fauna Silvestre), Forestal Rio Piedras SAC and Conservation International. Field staff of these institutions georeferenced individual trees in the company of the respective Brazil nut harvesters who were systematically asked to provide their recollection of the average productivity of each individual tree. For the vast majority of trees these data were complemented with DBH and height measurements, and a description of each tree’s phytosanitary condition, by indicating whether a tree was infested by lianas, had broken branches or holes in its trunk, or showed evidence of termite nests, wound exudate or tumours. The seed production estimates, DBH and height measurements and phytosanitary characterization data of individual trees used in this study were collected during one single field mission. Brazil nut seeds are harvested by cracking open the lignified capsular fruits (pyxidia) with a machete after these have fallen on the ground. Individual seeds are also protected by wooden testa, but these are not opened in the field and harvesters expressed seed production weight as multiples of ´latas´ (tin cans) which contain approximately 11.66 kg of fresh in-shell seeds. We compiled a dataset containing 135,528 georeferenced Brazil nut trees (S1 File) which had diameters at breast height (DBH) ≥10 cm from 418 of the approximately 1,200 Brazil nut concessions in Madre de Dios, Peru (Fig 1), which were largely collected by the above institutions in the period between 2003 and 2007.

A limitation of this dataset is the low precision of georeferences of numerous individual trees, related to the use of low-precision GPS equipment, further complicated by difficulties to capture satellite transmissions under the forest canopy. Furthermore, some coordinates were obtained through the use of compass and measuring tape, using points with known coordinates as references. However, as most of the spatial layers (climate and soil) we used for extracting environmental data had maximum resolutions of 30 arc seconds (~1km at the equator) and are themselves based on interpolated data (for further details see S2 File), imprecision issues are not expected to excessively influence the analyses carried out here.

In May 2012, we participated in a group interview organized by CIFOR (Center for International Forestry Research) with 8 female and 21 male concession holders of the Brazil nut association ASCAL to identify (through free listing) the variables that, according to the participants, influence the productive potential of Brazil nut trees (data used with permission). We also organized individual interviews with 53 additional concessionaires (12 female and 41 male) and asked them to free list the variables they believed influence seed production in Brazil nut. We did not obtain the approval of an Institutional Review Board or ethical committee mainly because of the nature of the interviews conducted. The purpose of the study was not to collect personal information of participants but rather aggregated data about the variables that influence the seed production of Brazil nut trees for the evaluation of our research hypotheses. We obtained oral consent of all participants, after explaining them that the goal of the interviews was to verify whether local people´s perceptions match or not with other scientific findings. Participant names, dates and places of interviews and oral consent were recorded in field notes. While we consider the collected data to classify as local or traditional ecological knowledge, most of it is publically available as anecdotal knowledge (and, for example, reported in local students' dissertations) and in our view has little to no chance of being misused. Actually some of the variables reported by the people may have been mentioned to them by the plethora of institutions or professionals working in the area, and it is not really possible anymore to confidently distinguish endogenously developed knowledge from knowledge introduced by outsiders. Participants were informed that the results of the interviews could potentially be published in aggregated format, and we intend to make available the findings of this study to concessionaires and Brazil nut harvesters during ongoing and future activities of the authors in the region.

### Comparison of Brazil nut harvesters´ estimates with field measurements

In a recent study Rockwell et al. [9] measured seed production over two years of 499 Brazil nut trees (DBH≥40 cm) from five different concessions in Madre de Dios which are also included in our dataset of 135,528 trees. To evaluate the degree of correspondence between these measurements and the estimates provided by Brazil nut harvesters, we matched individual trees in the two datasets based on the correspondence between their geographical locations and measured diameters. For this, we used only the 457 trees in the Rockwell et al [9] dataset with unique coordinates and considered only trees whose diameters and locations could be matched with a 10% error margin and precisions of 10m, 20m and 40m, respectively. As the mean distances between Brazil nut trees sampled by Rockwell et al [9] varied between 57 and 81m, we expected the probability of matching the same trees in the two datasets to be inversely correlated with the degree of correspondence between their geographical locations.

### Statistical analysis

We validated the correspondence between seed production estimates of individual trees by Brazil nut harvesters and measurements carried out by Rockwell *et al* [9] by means of linear regression models. Normality of the model residuals was confirmed by a Shapiro-Wilk test (P>0.10) and residuals did not show evidence of spatial autocorrelation (confirmed by means of autocorrellogram constructed in *ncf* package for R [15,16]).

We assessed the nature of the relationships between Brazil nut seed production estimates and tree size variables (height, canopy position and size, DBH and aboveground biomass), distances to the three nearest conspecific neighbours, geophysical, edaphic and climatic variables with those reported in literature and/or by Brazil nut harvesters by means of basic and mixed generalized linear and additive models (GLMs and GAMs, respectively). We constructed the models using the Poisson distribution, given that the production estimates of Brazil nut trees were expressed as multiples of tin cans (~ 11.66 kg each) and hence show strong similarities with count data. To account for overdispersion (<3 in all cases), we corrected the standard errors using quasi GLM and GAM models, where the variance is given by the product of the mean and the dispersion parameter. For the additive models (implemented in *mgcv* package for R [17]) we used thin plate regression splines and cross-validation to estimate the optimal amount of smoothing. The presence of positive spatial autocorrelation in model residuals (assessed by means of case-specific autocorrellograms), was accounted for by including the concession where each tree was sampled as a random effect variable. GLMMs were implemented using Penalized Quasi-Likelihood in *MASS* package for R [18]. As this approach does not allow calculating (pseudo) R^2^ values or the proportion of the null deviance explained by the model, we additionally carried out Spearman regressions to complement GLMM results. To enhance interpretation of figures, response variables and fitted values were transformed to seed production estimates expressed as kilograms.

Each of the individual GLMs and GAMs is expected to explain only a tiny portion of the variability in seed production estimates for three main reasons. First, seed production of individual Brazil nut trees is influenced by the interplay of multiple genetic, physiological, demographic, phytosanitary, climate, weather, edaphic and anthropogenic variables, implying that single variables are likely to explain only a small fraction of the variation in seed production. Second, several of the explanatory variables (climate, soil, terrain) may have low precision as they themselves are based on interpolations. And third, the seed production estimates are expressed as multiples of the content of tin cans (~ 11.66 kg), and were provided by a large number of people, whose individual estimates are expected to show substantial variation in terms of quality. However, in line with the findings of Steinke et al. [5], we expect the averages of the pooled estimates to approximate true patterns between explanatory variables and the seed production of individual Brazil nut trees. We consider a pattern as significant if the average increase or decrease in seed production estimates along the regression line and across the range of an explanatory variable captured by our data is at least 10% of the average seed production estimate of all trees in our dataset (30.6 ± 26.9 (SD) kg per tree), i.e. ~3kg.

For comparisons of seed production estimates of Brazil nut trees across different categorical variables (phytosanitary conditions, growing close or not to rivers and roads, in and outside indigenous territories and protected areas), we used non-parametric Wilcoxon tests due to violation of the homoscedasticity assumption for the vast majority of comparisons, ruling out the use of t-tests.

## Results

**Fig 2:**
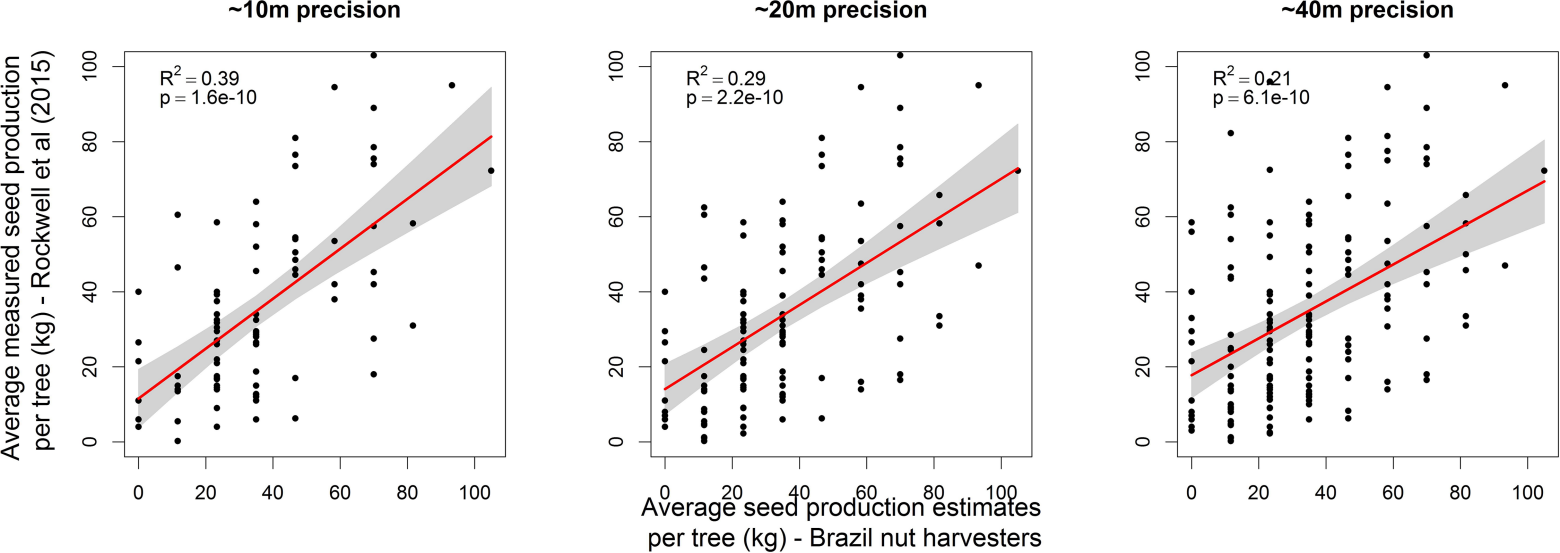
Relationship between average seed production of individual Brazil nut trees measured over two years by Rockwell et al. [9] and seed production estimates by Brazil nut harvesters. Only trees that could be matched with a precision of ~10m (n = 86), ~20m (n = 121) and ~40m (n = 166), respectively, and had a similar diameter (10% error margin) were considered out of a total of N = 457 trees with unique coordinates measured by Rockwell et al. [9]. The regression lines are in red and 95% confidence intervals are indicated by grey polygons.

Correlations between the harvesters’ seed production estimates of trees in our dataset and average values measured by Rockwell et al. [9] are consistently positive, but yield decreasing R^2^ values as the degree of uncertainty of the match between trees in the two datasets increases (Fig 2).

**Fig 3:**
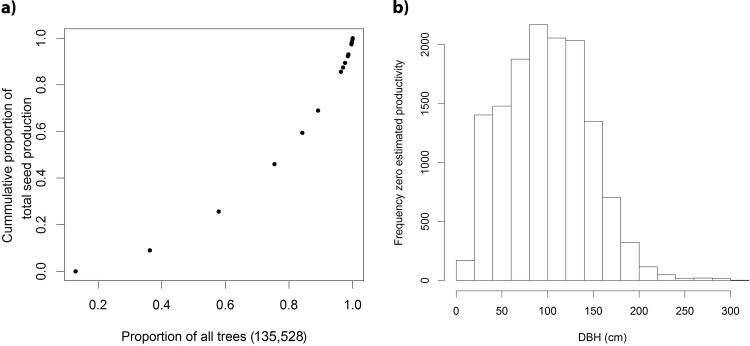
a. Cumulative contribution to overall estimated seed production of all 135,528 trees in our database ordered by their individual production estimates b. Diameter distribution of trees that were said by Brazil nut harvesters to never or hardly ever produce seeds.

The cumulative contribution to the overall estimated seed production of trees in our database ordered by their individual production estimates showed an exponentially increasing trend (Fig 3A). A substantial part (13%) of all trees were claimed by the harvesters to never or hardly ever produce any fruits, while 25% of the trees were responsible for more than half of the total seed production. The vast majority of trees with zero estimated productivity had diameters smaller than 150 cm DBH (Fig 3B).

Table 1 lists the main variables that influence (either positively or negatively) the productive potential of Brazil nut trees as identified in the literature and during ethno-ecological interviews with Brazil nut concessionaires. The variables reported by concession holders were: soil type, soil drainage (*terra firme* vs flooded), precipitation, the number of branches, canopy form, closeness to conspecific neighbour, presence of companion species (whose presence is believed to increase fruit production, including *Cariniana spp*., *Ceiba pentandra*, *Copaifera sp*., *Hevea brasiliensis*, *Nectandra longifolia*; *Tabebuia serratifolia* and *Tetragastris altissima*), health status, liana infestation, fruit predation by parrots, infestation by leaf cutter ants, strangler epiphyte infestation, imminent mortality (trees about to die produce more), being a *macho* tree (which never produce), pollinator presence, seed production history of a tree, fire and smoke produced by neighbouring land uses and gold mining.

**Table 1:** Relations between seed production of Brazil nut trees and a number of phenotypic, phytosanitary and environmental variables described in literature and/or reported by Brazil nut concessionaires and harvesters, compared with those obtained from seed production estimates sourced from harvesters in Madre de Dios.

	Literature references and traditional/local knowledge				Productivity estimates this study		
	variable	Correlation	observations	Source/ Reference	variable	Correlation	observations
**Tree size**	**DBH**	unimodal	Highest production by trees in the middle diameter range (100–150 cm)	[11,19]	DBH (Fig 4D)	unimodal	Highest production by trees in middle diameter range (125–250 cm)
		+	Production positively correlated with tree diameter	[10,12]			
	**Tree height**	+	Taller trees tend to produce more	[8]	Tree height (Fig 4C)	+	Total tree height
**Canopy exposure and** **light availability**	**Crown diameter**	+	Bigger crown diameters are associated with higher productivity	[9]; Leigue Gómez and Boot cited in [7]	Crown height (Fig 4B)	+	
	**Crown** **form**	+	Crown with complete or irregular circular forms tend to produce more	[9–12]		NA	
		+	Trees with more symmetric canopies produce more	interviews		NA	
	**Number of branches**	+	Trees with higher numbers of branches produce more	interviews		NA	
	**Crown** **position**	+	The higher the crown position the higher the productivity	[10,12]	Crown position (Fig 4A)	+	Height until first branch
	**Light** **exposure**	+	Higher light availability/canopy exposure is associated with higher productivity	[7]	Vicinity of road (see text)	+	Closeness to road is expected to correlate with increased light exposure
**Pests, diseases and phytosanitary state**	**Liana** **load**	_	Liana presence reduces production	[7,9,19,20]	Liana load (Fig 5F)	_	
		_	Trees with liana load or hemiepiphytic strangler infestation produce less	interviews			
	**Damage or disease**	_	Trees presenting damage (wounds) or disease produce less	interviews	Broken branches (Fig 5A)	_	
					Wound exudate (Fig 5B)	_	
					Hole in trunk (Fig 5C)	_	
					Tumour (Fig 5E)	NS	
	**Trees close to dying**	+	Trees that are close to dying produce more	interviews		NA	
	**Parrots**	_	Parrots eat immature fruits	interviews		NA	
	**Leafcutter** **ants**	_	Attacks by leafcutter ants lower seed production	interviews		NA	
**Genetics and reproductive biology**	**Good production history**	+	Trees with good production in the past are likely to produce well in the future	interviews		NA	
	**Seed production concentrated** **in limited proportion of trees**	+	Most trees produce small quantities of fruits and some 25% of the trees are responsible for the large majority of seed production at stand level	[7,11]	Seed production concentrated in limited proportion of trees (Fig 3A)	+	25% of all trees were responsible for 54% of the overall seed production estimates
	**Macho tree**	_	Some trees (locally called macho trees) never produce	interviews	Macho tree (Fig 3A)	_	13% of trees had zero estimated production
			A substantial proportion of trees does not produce in a given year	[8]: 7.5%; PROMAB 1999, cited in [7]: 16%; [11]: 14% [10]: 19%			
	**Presence of pollinators**	+	Production better when trees grow close to forest, compared to plantations outside of forest	[7,21]		NA	
		+	In ecosystems that are natural habitat of pollinator bees production tends to be higher	interviews			
	**Closeness to conspecific trees**	+	Trees that grow closer to conspecific trees produce more	interviews	Closeness to conspecific trees (Fig 6)	unimodal	Negative correlation between nearest conspecific distances and seed production only applies to trees at conspecific distances of 100m or more
	**Isolated trees**	_	Isolated trees tend to produce less	interviews	Isolated trees (Fig 6)	-	Trees that are at large minimum conspecific neighbour distances tend to produce less
	**Closeness to companion species**	+	Brazil nut is dependent on other plant species for pollination and fruit production	[21,22]		NA	
		+	Vicinity of companion species enhances productivity by attracting pollinators	interviews			
**Climate and weather**	**Annual precipitation**	+	Lower fruit production in years with lower precipitation (e.g. El Niño years)	[7,11]	Annual precipitation (S3D Fig)	+	
		+	More rain generally means higher seed production, but rain is particularly important from March to May	interviews			
	**Precipitation dry season**	+	Highly significant correlation between rainfall during previous five months of dry season (May-September) and fruit production	[11]	Precipitation of driest quarter (S3F Fig)	+	
		+	Lower precipitation during the dry season means lower productivity	interviews			
	**Wind**	-	Strong winds may decrease productivity	interviews		NA	
**Soil and terrain variables**	**CEC**	+		[11]	CEC (S2E Fig)	NS/+	GLMM and Spearman suggest non-significant and positive correlation, respectively
	**Extractable P**	-		[11]		NA	
	**Dark earth**	+	Higher production in dark earth soils	interviews	Organic matter (S2D Fig)	+	Dark earth is usually associated with higher concentration of organic matter
	***Terra*** ***firme***	+	Trees in inundated areas produce less	interviews	Elevation (S1A Fig)	+	As the study area is flat, higher elevation tends to correlate with *terra firme* soils
					Distance to river (see text)	+	Trees at higher distances from rivers are more likely to be located on *terra firme*
**Anthro-pogenic disturbance**	**Harvest intensity**	+	Intensively used Brazil nut stands are more productive than moderately used ones	[23]	Harvest intensity (see text)	+	Productivity of trees higher in concessions than in protected areas
		_	In areas with lower human intervention trees tend to produce more	interviews			
	**Fire and** **smoke**	_	Smoke produced by burning of forest and swiddens scares away pollinators, leading to lower seed production	interviews		NA	
	**Gold mining**	_	Mining operations lower productivity	interviews		NA	
	**Logging**	_	Logging may lower seed production	[9]		NA	

Significant (at p<0.05) positive and negative correlations are indicated with “+” and “-”, respectively (NS = not significant, NA = not applicable).

Where feasible we gauged the nature of the relationships between each of these variables and harvesters’ seed production estimates of individual Brazil nut trees in our dataset and compared these with findings from literature and claims by concessionaires about the effects of different variables on Brazil nut seed production. We found clear matches between trends in our data and those reported in literature and during interviews in nearly all cases (Table 1). Our finding that estimates of average seed production of trees in protected areas were significantly lower than of trees from Brazil nut concessions does support Scholes and Gribel’s [23] assertion that intensively used Brazil nut stands are more productive than moderately used ones, but contrasts with opposite claims by Brazil nut concession holders. Only one concessionaire who we interviewed predicted our finding but attributed lower seed production in protected areas to the fact that those trees grow in suboptimal habitat conditions for Brazil nut.

**Fig 4:**
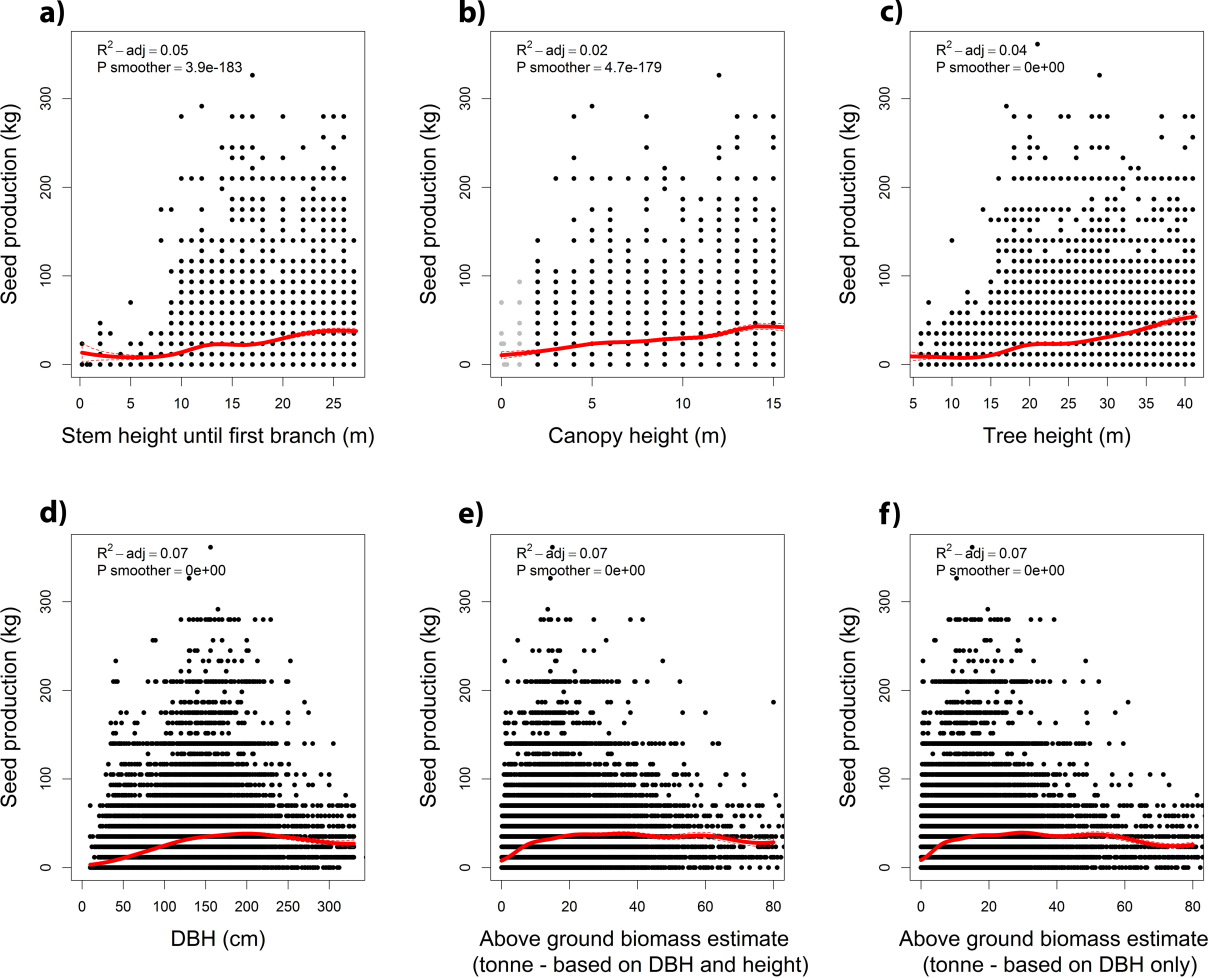
**Relations between estimated seed production of Brazil nut trees and (a) stem height to the first branch (crown position); (b) crown height; (c) total tree height; (d) DBH; (e) above ground woody biomass based on DBH and height measurements; and (f) above ground woody biomass based on DBH only.** Red lines represent GAMM smoothers. Dashed lines show 95% confidence intervals.

All variables related to the size of trees correlated positively with seed production estimates (Fig 4). However, while trends of monotonically increasing seed production were observed for height variables (Fig 4A–4C), unimodal relationships were found for stem diameter and aboveground wood biomass estimates (Fig 4D–4F).

**Fig 5:**
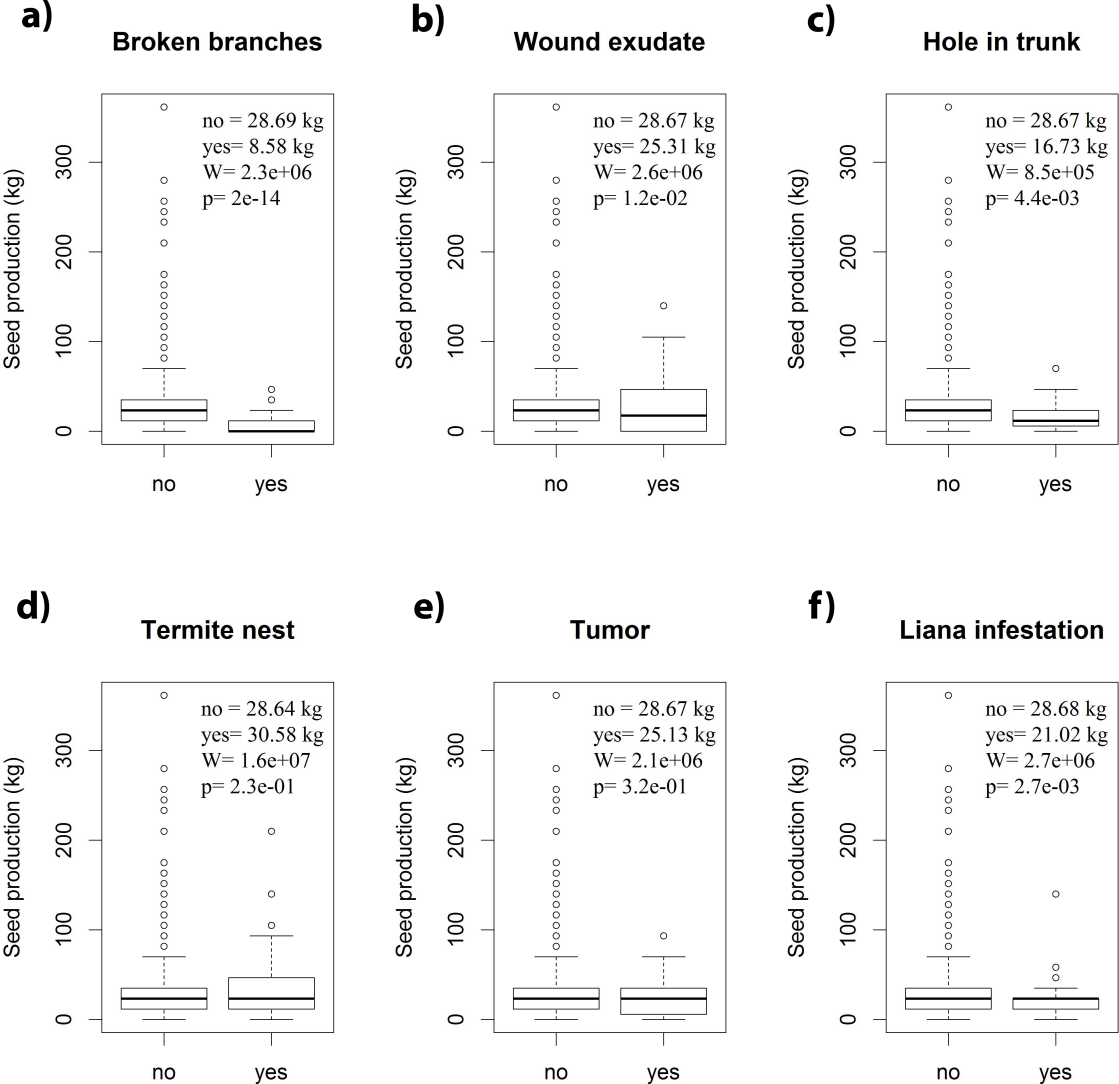
**Boxplots comparing Brazil nut seed production estimates between trees with and without (a) broken branches; (b) evidence of wound exudate; (c) holes in their trunks; (d) presence of termite nests; (e) presence of tumours; (f) liana infestation.** Statistical comparisons are based on Wilcoxon tests (n = 55,644).

Brazil nut trees in our dataset had significantly lower seed production estimates when they either had broken branches, holes in the trunk, evidence of wound exudate or liana infestation (Fig 5A–5C and 5F). The presence of termite nests or tumours did not influence seed production estimates (Fig 5D and 5E).

Seed production estimates correlated positively with the elevation of a tree´s growth site and negatively with slope (p<<0.001; S1A and S1B Fig). We did not find an effect of aspect (direction of the slope) on productivity (S1C Fig). Trees growing in 7.5 arc second grid cells crossed by rivers yielded lower average seed production estimates (26.97kg/tree) than trees at further distances from rivers (30.47kg/tree, Wilcoxon W = 5.5e108; p = e-33). An opposite trend was found for trees growing in 7.5 arc second grid cells crossed by roads (32.26kg/tree), compared to trees at further distances (30.41kg/tree, Wilcoxon W = 1e108, p = 0.016). Seed production estimates of Brazil nut trees correlated positively with expected clay and silt content, the organic carbon fraction and negatively with sand content and pH of soils at growth sites (p<0.01; S2 Fig). The relation between the expected cation exchange capacity (CEC) of the soil at a tree´s growth site and estimated seed production was not significant according to our GLMM, but nonetheless yielded a highly significant positive Spearman correlation coefficient (p<<0.001; S2 Fig).

We found a consistent trend of lower estimates of seed production for Brazil nut trees growing at sites with higher air temperature levels (p<<0.001; S3A–S3C Fig). Higher production estimates were documented at sites with higher precipitation (p<0.05; S3D Fig). This effect was strongest for the amount of precipitation in the driest quarter and the driest month of the year (p<0.001; S3E and S3F Fig).

**Fig 6:**
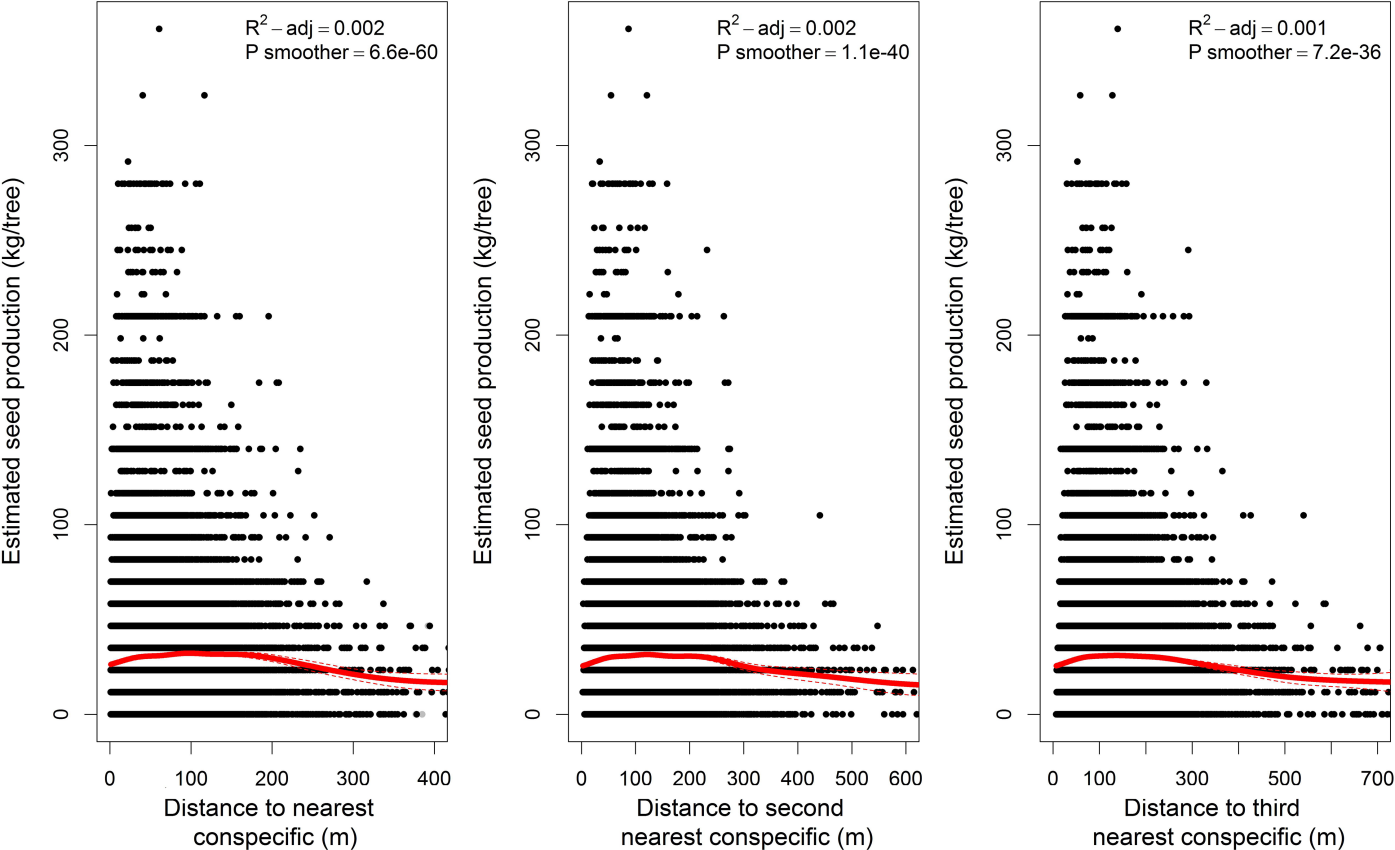
Relation between estimated seed production of Brazil nut trees and the distance to the three nearest conspecific trees. The solid red lines represent GAM smoothers. Dashed lines show 95% confidence intervals.

Brazil nut trees growing closer to conspecific trees tended to yield higher seed production estimates than more isolated trees (Fig 6). However, the fine-scale relationship between the distance from a tree to its nearest conspecific neighbours and seed production estimates was closer to an unimodal than a linear one, with both very short and long distances yielding lower productivity estimates than intermediate ones.

Average productivity estimates tended to be lower for trees growing in protected areas (22.38kg/tree) than in Brazil nut concessions (30.89; Wilcoxon W = 7.6e8, p = 2.1e-283). We found slightly lower average productivity estimates for trees growing inside (28.23kg/tree) compared to outside indigenous territories (30.37kg/tree; Wilcoxon W = 4.4e8, p = 3.2e-4).

## Discussion

Two main lines of evidence validate the usefulness of seed production estimates of Brazil nut trees as reliable proxies for field measurements. First, we found positive correlations between Brazil nut harvesters´ production estimates and the field measurements collected by Rockwell et al. [9] for individual trees (Fig 2). Second, we found matching patterns for the relationships between seed production estimates and all explanatory variables tested or described in literature (Table 1). Furthermore, all patterns, including those not confirmed in literature, make biological sense, thus providing support for the reliability of seed production estimates. As expected, most variables explained only minor fractions of the variation Brazil nut seed production estimates. However, the difference in average seed production of trees at opposite ends of the ranges of all variables was in the order of 5-10kg- well above the minimum threshold of 3kg specified in the methods section (i.e. 10% of the average Brazil nut seed production estimates). In what follows we discuss the different variables tested.

### Phenotypic variables and light exposure

We found that average production estimates increased with stem and crown height variables. This corroborates previous experimental research which related these variables to increased light exposure which in turn is believed to positively influence seed production [7–12]. Accordingly, we did not find an effect of the direction of the growth site slope (aspect) on Brazil nut productivity estimates (S1C Fig). This was expected, as most productive trees are emergent and that fact that owing to the closeness of the study area to the equator, the sun stands almost perpendicularly above the forest canopies at noon. However, lateral light regimes might influence fecundity, as suggested by the higher average seed production estimates documented for trees growing in the vicinities of roads, compared to trees at further distances.

The unimodal relationships we found between seed production estimates and variables related with wood biomass are much less documented. Some studies found positive correlations [10,12] between a tree´s DBH and its average seed production, but to the best of our knowledge only Kainer et al [11,19] reported a maximum of seed production in the middle diameter classes (100–150 cm DBH). The peak in our production estimate data similarly started around 100 cm DBH, but reached its true maximum between 125 and 250 cm DBH. This discrepancy may be because of the much smaller sample size of the studies of Kainer et al [11,19], combined with the fact that maximum tree sizes were lower in their study area (their biggest tree was 210 cm DBH). Interestingly, the maxima of estimated seed production of trees occurred roughly in the 100–200 cm diameter class, thus preceding the peak in average seed production estimates (red line in Fig 4D). This is because the vast majority of trees with zero estimated productivity were less than 150 cm DBH (Fig 3B). Peaking seed production in middle diameter classes is paralleled by the unimodal relation we found between the aboveground biomass estimate of a tree and its estimated seed production. There is evidence that some tree species produce most seed in middle age [24], followed by decline and senescence. While the relation between tree diameter and age in Brazil nut trees individuals can be highly variable, overall it seems to abide to an approximately linearly increasing trend [25].

### Phytosanitary variables

Liana load is one of the best studied phytosanitary variables that negatively affect seed production of Brazil nut trees [7,9,19,20]. Our results not only corroborate previous studies, but also support assertions of Brazil nut harvesters that trees with broken branches, holes in their trunks or showing the presence of wound exudate tend to produce less (Fig 5). The negative effect of heavy winds on seed production identified by Brazil nut harvesters may be related to the association between wind and the incidence of broken branches.

### Genetics and reproductive biology

Our data suggest that genetics influence seed productivity, at both individual and population levels. Individual-level genetics might explain why some trees never or hardly ever produce seeds (e.g. because of incompatibility issues) while others produce very high quantities of seeds year after year (e.g. by genetic superiority). Indications of the potential impact of population-level genetics on seed production are apparent in the relationship between the recorded seed production of a tree and the distance to its nearest conspecific neighbors. Our findings support the observation of concessionaires that seed production tends to increase in Brazil nut trees that grow closer to conspecific neighbours. This is called the “Allee” effect [26] and is related with effects of pollen limitation and increased selfing rates in more isolated trees [27]. It has been described for numerous tree species [28,29], particularly in predominantly outcrossing ones such as Brazil nut [30]. However, this effect only applied to Brazil nut trees growing at nearest conspecific distances higher than 100 m, whereas trees growing at very short distances of conspecific neighbours tended to yield slightly lower production estimates than trees at intermediate distances (Fig 6). Considering the low population density of Brazil nut in the study area (approximately one tree per two hectares), this may be due to the fact that close conspecific neighbours are more likely to be genetically related (siblings or half siblings). Crosses between conspecific neighbours with high local kinship result in biparental inbreeding which may increase seed abortion rates [31], and hence lower seed set of trees [32]. Further research is needed to confirm or refute this hypothesis.

### Terrain and soil variables

Our results suggest that Brazil nut trees tend to produce more when they grow on higher lying, flat soils. Higher elevations in Madre de Dios are likely to correlate with *terra firme* soils (i.e. Brazil nut’s preferred habitat [33]) and lower elevations with floodplains/varzeas and proximity to rivers. This was confirmed by our finding that trees growing close to rivers yielded lower average seed production estimates than trees at further distances. Clay and Clement [34] argued that Brazil nut typically grows on well-drained ultisols and oxisols which have low pH. Accordingly, most of the Brazil nut trees in our dataset grew on soils with expected pH below 5 and there was a tendency for trees in more acid soils to yield higher production estimates (S2F Fig). In line with expectations, higher estimates were found for trees in soils with higher predicted organic carbon content (S2D Fig), which could also explain why Brazil nut harvesters reported that Brazil nut trees produce more in darker soils. The finding of Kainer et al [11] that higher CEC values tended to be associated with higher fecundity was only partly supported by our data (results Spearman correlation, but not GLMM) (S2E Fig). This does not necessarily mean our data were unable to convincingly detect this pattern. CEC is often mistakenly considered a proxy of soil fertility because soils of any given CEC can differ greatly in nutrient availability in Amazonia [35]. Further research is needed to assess the extent to which the nature of the relation between soil CEC and Brazil nut seed production is region or site specific.

### Climate and weather variables

Moderate temperature regimes seem to positively influence seed production of Brazil nut trees. Higher temperatures during the warmest periods of year were associated with lower production estimates (S3A–S3C Fig). Particularly the latter observation is troublesome in light of climate change which is expected to result in rising annual temperatures and increases in the frequency of climate extremes, and hence might lead to lower production levels. Precipitation correlated positively with production estimates, particularly so in the drier periods of the year (S3D–S3F Fig), thus confirming previous studies [7,11]. The fact that this trend was strongest for precipitation in the driest month to some extent contradicts the common -but previously challenged [36]- assumption that Brazil nut needs at least two dry months (<60mm) for development and growth [37]. Anecdotal evidence from Madre de Dios that a combination of high temperatures and droughts in 2005 and 2010 led to massive flower abortions (William Moreno, per. communication), suggests that temperature and precipitation interact in their impact on productivity, which requires further research.

### Anthropogenic variables

There has been a longstanding debate about the sustainability of harvesting Brazil nut seeds from natural stands with opposing views defended by different scholars [23,38]. While our data do not provide a firm stand in this debate, the fact that average seed production estimates of Brazil nut trees growing in protected areas were significantly lower than of trees from Brazil nut concessions, may support the hypothesis of Scoles and Gribel [23] that more intervened stands could be more productive. Brazil nut trees from protected areas are expected to be exposed less to anthropogenic disturbance compared to Brazil nut concessions where modifications of the understory and hunting of the main natural seed disperser (thus freeing more abandoned scatter hoards for germination) may promote natural regeneration and possibly seed production at stand level [23]. Alternatively, lower productivity estimates may reflect the fact that protected areas are located on suboptimal land for Brazil nut, as claimed by one of the Brazil nut harvesters we interviewed. A third scenario is that Brazil nut harvesters may have more difficulties to estimate seed production of individual trees in protected areas which they are allowed to enter only during the harvest season. It is widely accepted in ethnobotanical literature that more intensive contact with plant resources enhances knowledge on their useful traits [39], and this may also apply to seed production estimates.

The reason why productivity estimates of Brazil nut trees from indigenous territories tended to be lower than from Brazil nut concessions outside these areas is less clear. Aside from the possibility that at least some indigenous territories may also be located on suboptimal land for Brazil nut, the more sporadic harvesting practices of indigenous groups, or mistrust of the state authority at the time of data collection, coinciding with the first effort to make detailed inventories of Brazil nut concessions, may have resulted in their underestimating of average seed production levels.

## Concluding remarks

Our findings provide strong support for the value of citizen science data for detecting trends in Brazil nut seed production as a function of anthropogenic, environmental and inherent tree variables. All the patterns we found either confirmed experimental research results and/or are in line with biological theory. The large quantity of crowdsourced data on Brazil nut may even allow detection of trends that are difficult to uncover without complex and expensive experimental field setups. Some studies from literature came to unexpected conclusions possibly related to the relatively small sample sizes used. For example, Wadt et al [10] found a positive correlation between crown vine load and seed production, which was called into question by later work [11,19].

More concretely, our results suggest that productivity estimates of Brazil nut trees and possibly other NTFP-producing species, on a case-by-case basis, may serve as a cost-effective alternative or complement to field measurements, at least in areas where harvesters have a tradition of collecting NTFPs from the same trees over multiple years or decades. Sourcing productivity estimates from harvesters through registers on an annual basis, would not only help to improve data quality, but also allow a more robust monitoring.

As expected, nearly all TEK on Brazil nut productivity was corroborated by our data. This is reassuring as Brazil nut concession holders, and NTFP harvesters at large, rely on their knowledge to guide the management of the trees upon which their extractive economies are based. Furthermore, it can help to identify variables that influence the productivity of Brazil nut trees but have not yet been the subject of experimental research and require more attention by the scientific community. Notably the effects on seed production of ecological relations between Brazil nut trees and associated fauna and flora are currently understudied. It is clear that the presence of pollinators has a direct relation with the effectiveness of seed set in Brazil nut whose allogamous flowers are pollinated by large bees [40]. However, the effect of the vegetation matrix on the presence of pollinators and hence Brazil nut productivity remains unclear. For example, both Brazil nut harvesters and research scholars [7,21] have argued that seed production is positively influenced by natural forest cover which is the preferred habitat of the Brazil nut pollinator bees [41], as well as by the presence of other plant species that are visited by these bees [22,42]. The smoke produced by vegetation burning has been cited by harvesters as negatively affecting pollinator presence and Brazil nut seed production, in line with findings elsewhere [43]. The fact that the flowering of the Brazil nut in Madre de Dios (December to February [44]) overlaps with the wettest period of the year when vegetation burning is minimal, might suggest that smoke and fire produced during other periods of the year may influence the bees’ presence, and possibly abundance, in the flowering season.

The direct effects of logging on Brazil nut have been the subject of recent studies [9,45], but some indirect effects may also be at play. Some of the concession holders we interviewed said that while parrots have always fed on unripe Brazil nut fruits, this trend has increased in recent years due to logging of other tree species they traditionally feed on such as *Dypterix* spp. Also the potential negative effects of leafcutter ants on seed production have not yet been experimentally quantified, which would allow assessing the need (or not) for the development of management practices.

While lacking irrefutable scientific proof for many of the ecological relationships mentioned above, management decisions can already be adjusted to incorporate this TEK, for example by banning the logging of timber species whose presence is believed to positively influence the presence of bee pollinators, or of species whose logging may affect the feeding behaviour of parrots that damage Brazil nut fruits.

## Supporting information

S1 File Raw Brazil nut data. (CSV)

S2 File Generation of explanatory variables used for predicting Brazil nut seed production estimates. (DOCX)

S1 Fig Relations between estimated seed production of Brazil nut trees and (a) elevation; (b) slope; and (c) aspect of the growth site, based on a 30m digital elevation model. Solid red lines represent predicted values of Penalized Quasi-Likelihood GLMM models. Spearman correlation statistics are given for reference.
(DOCX)

S2 Fig Relations between estimated seed production of Brazil nut trees and (a) sand; (b) clay; and (c) silt content; (d) organic carbon content; (e) cation exchange capacity and (f) pH of soil at growth site. Solid red lines represent predicted values of Penalized Quasi-Likelihood GLMM models. Spearman correlation statistics are given for reference.
(DOCX)

S3 Fig Relations between estimated seed production of Brazil nut trees and (a) annual mean temperature; (b) maximum temperature of the warmest month; (c) mean temperature of the warmest quarter; (d) annual precipitation; and precipitation of the (e) driest month and (f) driest quarter at the growth site. Solid red lines represent predicted values of Penalized Quasi-Likelihood GLMM models. Spearman correlation statistics are given for reference.
(DOCX)
