# Supplementary material for: NTFP harvesters as citizen scientists: Validating traditional and crowdsourced knowledge on seed production of Brazil nut trees in the Peruvian Amazon
Source: PLoS One. 2017 Aug 24;12(8):e0183743. doi: 10.1371/journal.pone.0183743 (PMC5570363; doi:10.1371/journal.pone.0183743)
Supplement: S2 File — (DOCX) [file pone.0183743.s002.docx]

**S2 File**

**Generation of explanatory variables used for predicting Brazil nut seed production estimates**

We extracted environmental data from globally available spatial layers at the highest possible resolution. For elevation and the variables produced from it (slope and aspect) we used a 30m digital elevation model. Climate and soil variables were obtained from WorldClim [1] and ISRIC-World Soil Information [2], respectively (30 arc second rasters in both cases). We considered a set of bioclimatic variables (bio1, 5, 10, 12, 14 and 17) and the following six major edaphic variables: organic carbon (ORCDRC), pH in H2O (PHIHOX), sand % (SNDPPT), silt % (SLTPPT), clay % (CLYPPT), Cation Exchange Capacity (CEC). For the edaphic variables we calculated a weighted mean across 0–5, 5–15, 15–30, 30–60, and 60–100 cm soil depth values in order to derive a single data value for 0–100 cm. We identified which trees were located within and outside 7.5 arc second grid cells being crossed by mapped rivers and roads. Some of the Brazil nut estimates in our dataset come from protected areas (Tambopata National Reserve and Bahuaja Sonene National Park) where some people have legal concessions, notably people affiliated to the Brazil nut association ASCART (Asociación de castañeros de la reserve Tambopata)^[[1]](#footnote-1)^. Indigenous groups also engage in commercial Brazil nut harvesting from their territories. Together, this permitted comparisons of average seed production of trees inside and outside protected areas and indigenous territories.

We used the equations from Chave *et al.* [3] to calculate stem-level above ground biomass (AGB). As there were more trees for which DBH measurements were available (120,314 trees) than total tree height (107,972), with only 85,730 trees overlapping, we estimated ABG based on DBH only, as well as based on DBH and total height measurements. We used an overall wood density value of 0.59 g cm^-3 (^^[[2]](#footnote-2))^.

**References**

1. Hijmans RJ, Cameron SE, Parra JL, Jones PG, Jarvis A. Very high resolution interpolated climate surfaces for global land areas. Int J Climatol. 2005;25: 1965–1978. doi:10.1002/joc.1276

2. Hengl T, de Jesus JM, MacMillan R a., Batjes NH, Heuvelink GBM, Ribeiro E, et al. SoilGrids1km — Global Soil Information Based on Automated Mapping. PLoS One. 2014;9: e105992. doi:10.1371/journal.pone.0105992

3. Chave J, Réjou-Méchain M, Búrquez A, Chidumayo E, Colgan MS, Delitti WBC, et al. Improved allometric models to estimate the aboveground biomass of tropical trees. Glob Chang Biol. 2014;20: 3177–3190. doi:10.1111/gcb.12629

1. http://www.v-c-s.org/sites/v-c-s.org/files/PIR_Tambopata_2010-2013.pdf [↑](#footnote-ref-1)
2. http://www.fpl.fs.fed.us/documnts/TechSheets/Chudnoff/TropAmerican/pdf_files/bertho1new.pdf [↑](#footnote-ref-2)
