# Supplementary material for: NTFP harvesters as citizen scientists: Validating traditional and crowdsourced knowledge on seed production of Brazil nut trees in the Peruvian Amazon
Source: PLoS One. 2017 Aug 24;12(8):e0183743. doi: 10.1371/journal.pone.0183743 (PMC5570363; doi:10.1371/journal.pone.0183743)
Supplement: S1 Fig — Relations between estimated seed production of Brazil nut trees and (a) elevation; (b) slope; and (c) aspect of the growth site, based on a 30m digital elevation model. Solid red lines represent predicted values of Penalized Quasi-Likelihood GLMM models. Spearman correlation statistics are given for reference. (DOCX) [file pone.0183743.s003.docx]

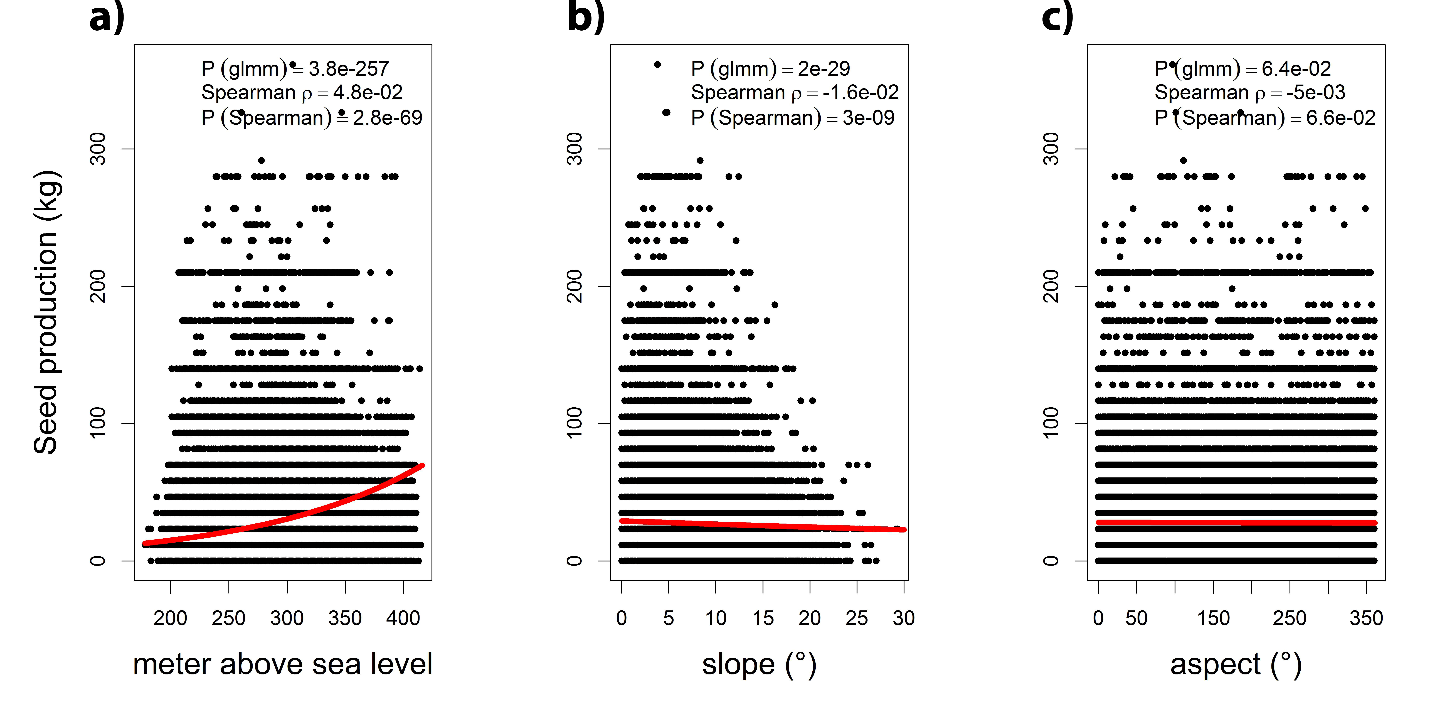


**Figure S1. Relations between estimated seed production of Brazil nut trees and (a) elevation; (b) slope; and (c) aspect of the growth site, based on a 30m digital elevation model. Solid red lines represent predicted values of Penalized Quasi-Likelihood GLMM models. Spearman correlation statistics are given for reference.**
