# Supplementary material for: NTFP harvesters as citizen scientists: Validating traditional and crowdsourced knowledge on seed production of Brazil nut trees in the Peruvian Amazon
Source: PLoS One. 2017 Aug 24;12(8):e0183743. doi: 10.1371/journal.pone.0183743 (PMC5570363; doi:10.1371/journal.pone.0183743)
Supplement: S2 Fig — Relations between estimated seed production of Brazil nut trees and (a) sand; (b) clay; and (c) silt content; (d) organic carbon content; (e) cation exchange capacity and (f) pH of soil at growth site. Solid red lines represent predicted values of Penalized Quasi-Likelihood GLMM models. Spearman correlation statistics are given for reference. (DOCX) [file pone.0183743.s004.docx]

**
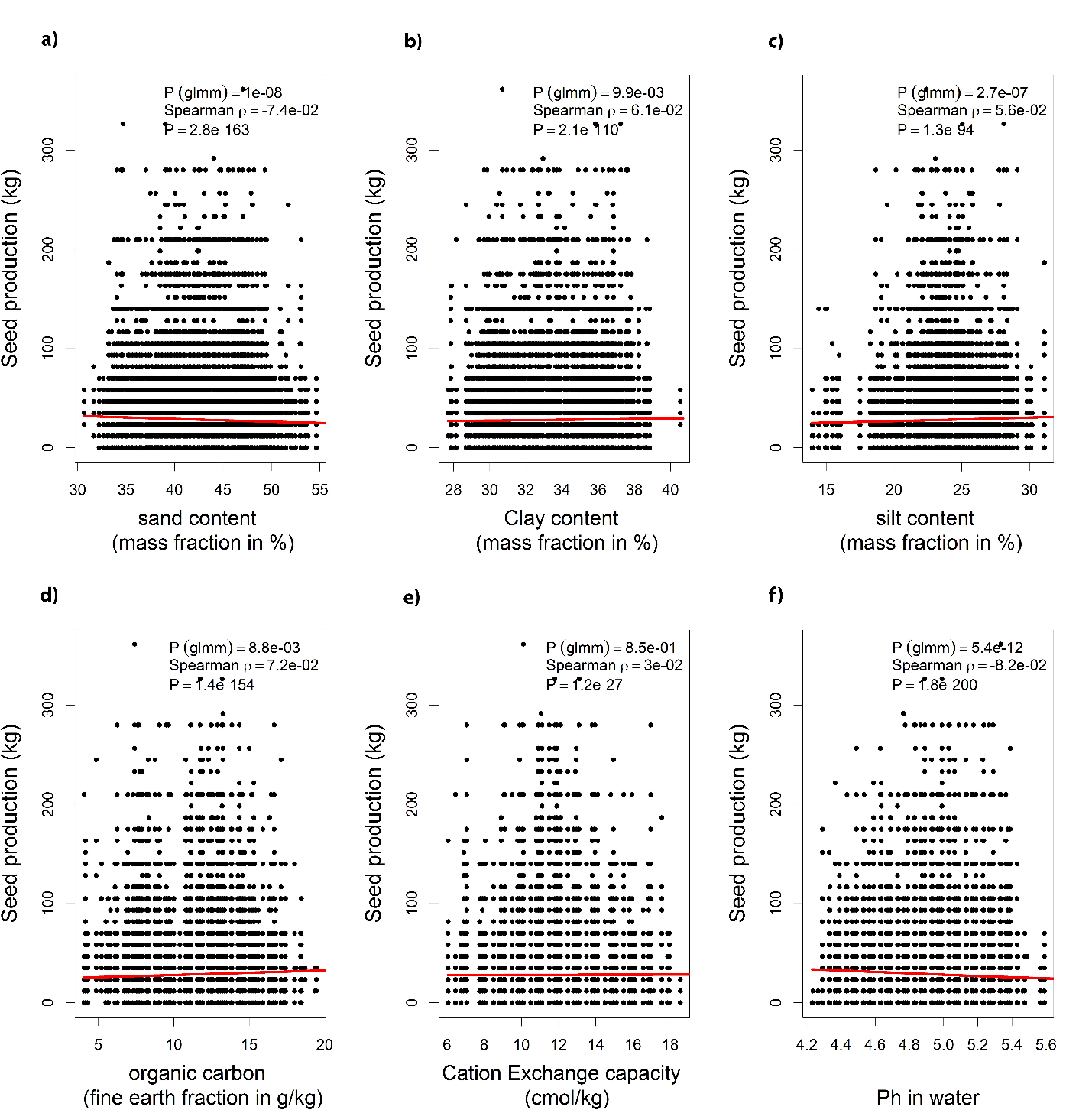
**

**Figure S2.** **Relations between estimated seed production of Brazil nut trees and (a) sand; (b) clay; and (c) silt content; (d) organic carbon content; (e) cation exchange capacity and (f) pH of soil at growth site, based on Hengl et al.** (2014)**. Solid red lines represent predicted values of Penalized Quasi-Likelihood GLMM models. Spearman correlation statistics are given for reference.**
