# Supplementary material for: NTFP harvesters as citizen scientists: Validating traditional and crowdsourced knowledge on seed production of Brazil nut trees in the Peruvian Amazon
Source: PLoS One. 2017 Aug 24;12(8):e0183743. doi: 10.1371/journal.pone.0183743 (PMC5570363; doi:10.1371/journal.pone.0183743)
Supplement: S3 Fig — Relations between estimated seed production of Brazil nut trees and (a) annual mean temperature; (b) maximum temperature of the warmest month; (c) mean temperature of the warmest quarter; (d) annual precipitation; and precipitation of the (e) driest month and (f) driest quarter at the growth site. Solid red lines represent predicted values of Penalized Quasi-Likelihood GLMM models. Spearman correlation statistics are given for reference. (DOCX) [file pone.0183743.s005.docx]

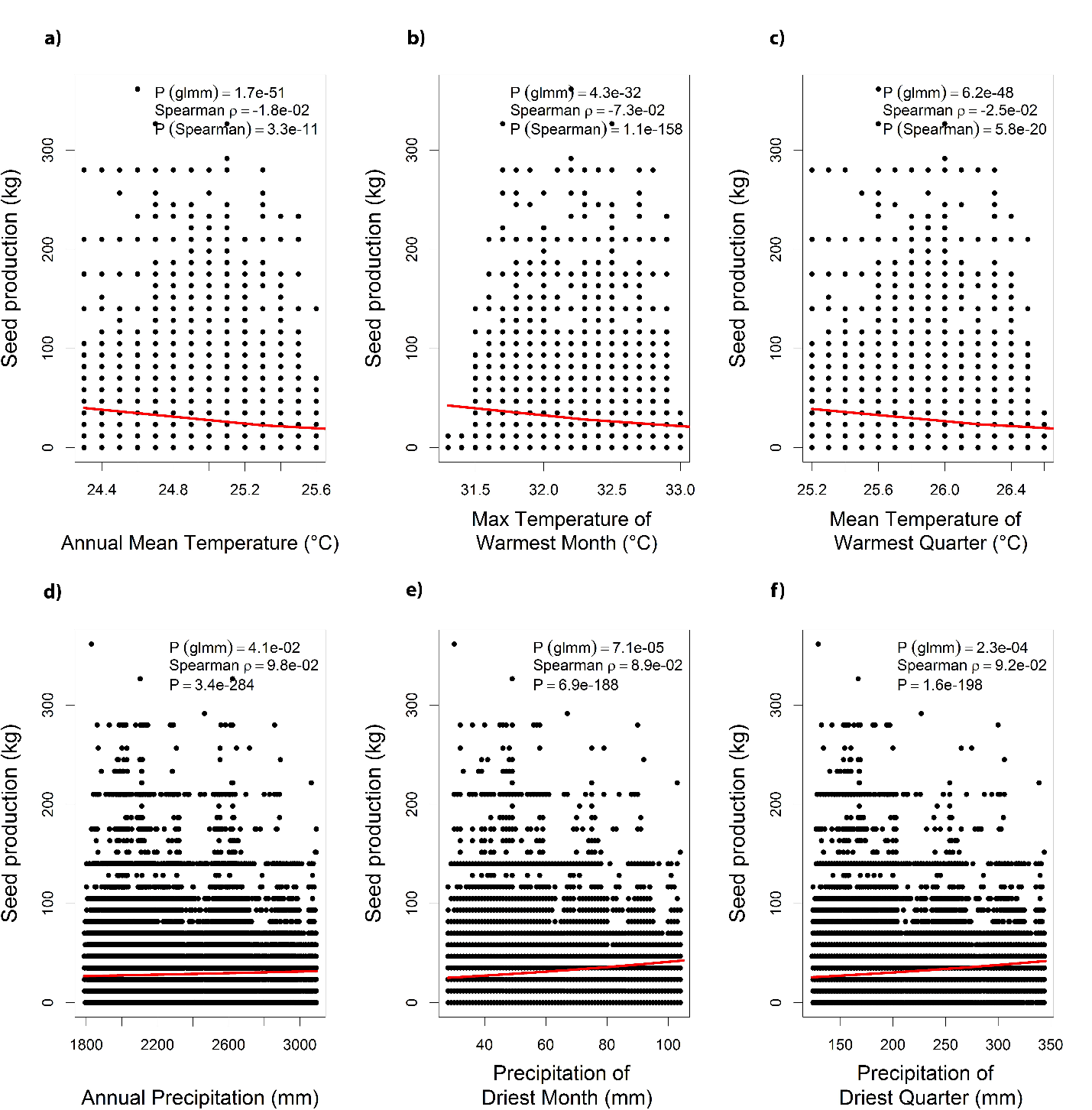


**Figure S3. Relations between estimated seed production of Brazil nut trees and (a) annual mean temperature; (b) maximum temperature of the warmest month; (c) mean temperature of the warmest quarter; (d) annual precipitation; and precipitation of the (e) driest month and (f) driest quarter at the growth site (based on Hijmans et al.** (2005)**). Solid red lines represent predicted values of Penalized Quasi-Likelihood GLMM models. Spearman correlation statistics are given for reference.**
